# Understanding health and care expenditure by setting – who matters to whom?

**DOI:** 10.1177/1355819620936721

**Published:** 2020-06-30

**Authors:** Jenny Shand, Stephen Morris, Manuel Gomes

**Affiliations:** 1 PhD Researcher, Institute of Epidemiology and Health Care, University College London, UK; 2 RAND Professor of Health Services Research, Department of Public Health and Primary Care, University of Cambridge, UK; 3 Associate Professor in Health Economics, Institute of Epidemiology and Health Care, University College London, UK

**Keywords:** health and care service utilisation, integrated health care systems, linked data sets

## Abstract

**Objective:**

To assess service use and associated expenditure across a range of care settings in one local authority in London, United Kingdom.

**Methods:**

An analysis of linked electronic health and council records of adults living in the borough of Barking and Dagenham, east London, for the financial year 2016/17. Unit costs were applied to individual service use to provide expenditure at an individual and population level for five settings of care. Population and expenditure volumes were compared for 32 possible combinations of service use.

**Results:**

The total expenditure for the cohort (114,393 residents) for 2016/17 was £180.1 million. Almost half (47%) of total expenditure was incurred by community care, social care and mental health services, with hospital care and primary care incurring, respectively, 35% (£63.3 m) and 18% (£32.6 m). The two most common combinations in terms of total population volume and expenditure were primary and hospital care, and primary, hospital and community care. Primary care was present in all combinations. Mental health service use accounted for just over a tenth of all expenditure in the borough, but using mental health services substantially increased mean expenditure per patient.

**Conclusions:**

A whole system perspective across all settings of care improves understanding of service user patterns. Setting-level analysis remains important, particularly for mental health users.

## Introduction

The growing prevalence of chronic diseases and an aging population places increasing demand for coordination across settings of care.^1,2^ Health care systems are investigating how to integrate services across care settings and pathways to transition from managing individual episodes to taking a population management perspective as a means to optimise resources and reduce unnecessary service use. There are many examples from high-income countries of how organisations are working to understand how best to design and deliver more integrated services and systems.^3,4^ Likewise, in England, the National Health Service (NHS) is moving from a patient group focus^5^ to holistic population based integration, with the creation of Integrated Care Systems (ICS) aiming to build on ongoing efforts to bring together separate organisations and care settings to promote population based planning and service delivery for defined geographies.^6,7^

There is growing use of linked datasets to build more complete understanding of population-wide health and care service utilisation, which can inform service integration efforts. To date, research into service utilisation has focused on individual settings, such as emergency hospital admissions,^8^ specific disease pathways, such as for diabetes,^9^ or specific population groups, for example people with multiple chronic conditions^10,11^ or older people.^12^ With improved reliability and availability of hospital and primary care datasets, research has increasingly focused on the last two groups, with emerging evidence finding a positive association between increasing age and/or number of conditions and service use.^13^ However, many such assessments do not consider the entire care pathway and exclude other care settings, in particular mental health, community services and social care and therefore only present part of the whole system perspective needed to help inform more integrated service delivery.

This descriptive study seeks to contribute to filling this gap by assessing service use across five settings of care (hospital, primary, community, mental health and social care) in one London local authority. Our focus is on understanding the expenditure overall and for each setting of care that is associated with different combinations of service use. This focus was informed by the need for service delivery to be financially sustainable.^7^ Clearly, our findings reflect population utilisation patterns in the specific local context within which the study is set but the approach we propose may provide useful insights for decision-makers and practitioners elsewhere seeking to improve service and system design to better meet the needs of their populations.

## Methods

We used a subset of a linked dataset created for a research programme in Barking and Dagenham (B&D), a local authority (borough) in east London, England, UK. Data from local government, health providers and purchasers of services (‘commissioners’) were linked at the individual level, providing a dataset that includes individual level demographic, socio-economic factors, markers of poor health and health and social care service use. An overview of the dataset is provided in the online supplement.

B&D is a densely populated urban borough, with 210,700 residents and high levels of deprivation, ethnic diversity and a young population compared to the rest of the country. Data included adults (age 19 or over) who were confirmed residents of B&D between April 2016 and March 2017 and who were registered with a B&D or Havering GP practice. Confirmed residents are defined as those who are listed on the national address register and on either another council dataset, the GP register or both.^14^ Children were excluded because of their different service utilisation patterns.^15^ Confirmed residents who had died or moved out of B&D before April 2017 were excluded as they had fewer than 12 months activity data.

### Health and social care expenditure

We included the following types of care: hospital services (accident and emergency (A&E) attendances, elective and non-elective inpatient stays and outpatient appointments); primary care contacts; prescriptions; community care contacts (home visits, appointments with community teams including nurses, pharmacists and allied health professionals); mental health services (inpatient stays and outpatient appointments); and social care (weekly care packages which included costs for crisis intervention, home care, supported living placements, residential and nursing home placements). The care expenditure was estimated from activity data. For hospital services, we used the Healthcare Resource Group (HRG) national tariff.^16^ To estimate primary care expenditure we used unit costs from the 2016/17 Unit Cost Health and Social Care for GP visit and non-GP visit costs.^17^ We further used local prescription data to calculate unit costs per prescription per GP practice that could be applied to individual prescription counts. For mental health and community services, we used data from the patient-level information and costing system from North East London NHS Foundation Trust (the local provider) to calculate unit costs for each care contact. Local government funded social care expenditure was obtained from the weekly billed cost for each care package provided (including in-year package revisions). We were unable to source data on self-funded social care, expenditure for equipment, transport and home adaptation. The total expenditure for the financial year was calculated by aggregating individual costs across all settings.

### Analysis

We created a binary measure for each setting of care, assigning the value one if the individual had any service use in that setting and zero otherwise. For each individual we counted the number of settings in which they incurred a cost. This provided information on the settings an individual incurred expenditure and the combination of settings. Figure A.1 in the online supplement shows the 32 possible combinations of service use across the five settings of care (including having no service use in any setting), with service use measured by expenditure. We identified those combinations that were most dominant in terms of total population and expenditure volumes. We also explored the combinations that were most prevalent for each individual setting by reviewing the proportion of service users and expenditure in that setting.

All analyses were conducted using Stata version 15.1.^18^

## Results

There were 201,393 records of confirmed residents of B&D on 1 April 2016. Of these, 52,968 were outside the age range and 18,754 had left the borough within the year (including deaths). We excluded a further 9980 as we were unable to match these with an NHS number; 5298 because they were registered with a GP practice outside of the borough. The final cohort included 114,393 adult individuals (in 58,929 households), equating to 77% of all adult residents in the borough at that time (Table 1). The total expenditure across the five care settings for the cohort in 2016/17 was £180.1 million, distributed as follows: 35% (£63.3 m) hospital care, 24% (£42.5 m) community care, 18% (£32.6 m) primary care, 12% (£22.0 m) social care services and 11% (£19.4 m) mental health services.

**Table 1.** Summary characteristics of the cohort and expenditure across the five settings, 2016/2017.

	Cohort		Total expenditure	
	n	%	Mean	SD
Age				
19–49	70,564	62	807	3749
50–64	25,827	23	1591	6194
65–75	9376	8	2794	8018
75–85	5751	5	5008	11,695
85+	2875	3	9436	16,697
Gender				
Female	60,463	53	1790	6514
Male	53,930	47	1334	5940
Ethnicity				
White	15,767	14	925	4083
Black or Black British	18,355	16	999	4284
Mixed	48,305	42	2351	8186
Other	2394	2	801	3083
Asian or Asian British	17,324	15	1041	4064
Unknown	12,248	11	1122	4763
Body Mass Index (BMI)				
Underweight	3628	3	1967	8624
Healthy	33,562	29	1443	6039
Overweight	35,658	31	1491	5923
Obese	27,846	24	1895	6531
Morbidly obese	4918	4	2677	8898
Unknown	8781	8	628	3914
Smoking status				
Non smoker	70,288	61	1432	5904
Former smoker	18,295	16	2403	7754
Smoker	23,476	21	1489	6199
Unknown	2334	2	254	1345
Chronic conditions				
Atrial fibrillation (AF)	1674	1	8551	16,649
Asthma	11,436	10	2445	7986
Cancer	3339	3	4967	10,925
Coronary heart disease (CHD)	3423	3	6108	12,331
Chronic obstructive pulmonary disease (COPD)	3423	3	6196	13,110
Dementia	740	1	18,351	23,181
Depression	9045	8	3277	9944
Diabetes	10,325	9	4207	10,648
Epilepsy	1566	1	5314	13,430
Heart failure	881	1	11,132	19,001
Hypertension	21,671	19	3555	9626
Hypothyroidism	4840	4	3664	10,569
Learning difficulty	694	1	15,932	26,981
Mental health	1452	1	9738	18,888
Palliative care^a^	291	0	15,474	24,236
Stroke	1849	2	8393	16,824
Benefits^b^				
None	80,337	70	1130	4492
Employment support allowance	6497	6	3291	10,085
Pension	5589	5	5274	13,139
Income support	3506	3	2218	9441
Job seekers allowance	2024	2	966	2419
Standard	16,440	14	1751	6929
Housing tenure				
Owner-occupied	60,411	53	1307	5092
Private rented	23,459	20	1193	5441
Social housing	29,554	26	2275	8220
Unknown	969	1	6185	13,382
Household occupancy				
1	14,362	13	3751	11,009
2 to 4	67,606	59	1416	5591
5 to 7	27,293	24	886	3899
8 to 10	4009	4	772	2522
11+	1123	1	2933	9081
Deprivation (2015 Index of Multiple Deprivation, national quintiles)^c^				
Quintile 3	8818	8	1342	5375
Quintile 4	40,873	36	1474	5752
Quintile 5	64,702	57	1671	6655

^a^Palliative care was included in the list of ‘chronic conditions’ as a marker of increased acuity. It is likely to be associated with increased service use, particularly community services.

^b^The benefits system in England provides financial support for those who are unemployed and looking for work. It also provides people with assistance if their earnings are low, if they have a disability, are bringing up children, are retired, care for someone or are ill. A weekly amount is paid by the government to the eligible individual, with the level varying according to their circumstances.

^c^The Index of Multiple Deprivation 2015 is the official measure of relative deprivation for small areas (or neighbourhoods) in England. It combines information from seven domain indices (which measure different types or dimensions of deprivation) to produce an overall relative measure of deprivation. All areas in England are then ranked from 1 (least deprived) to 32,844 (most deprived).

### Individual expenditure profiles by care setting

**Figure 1.**
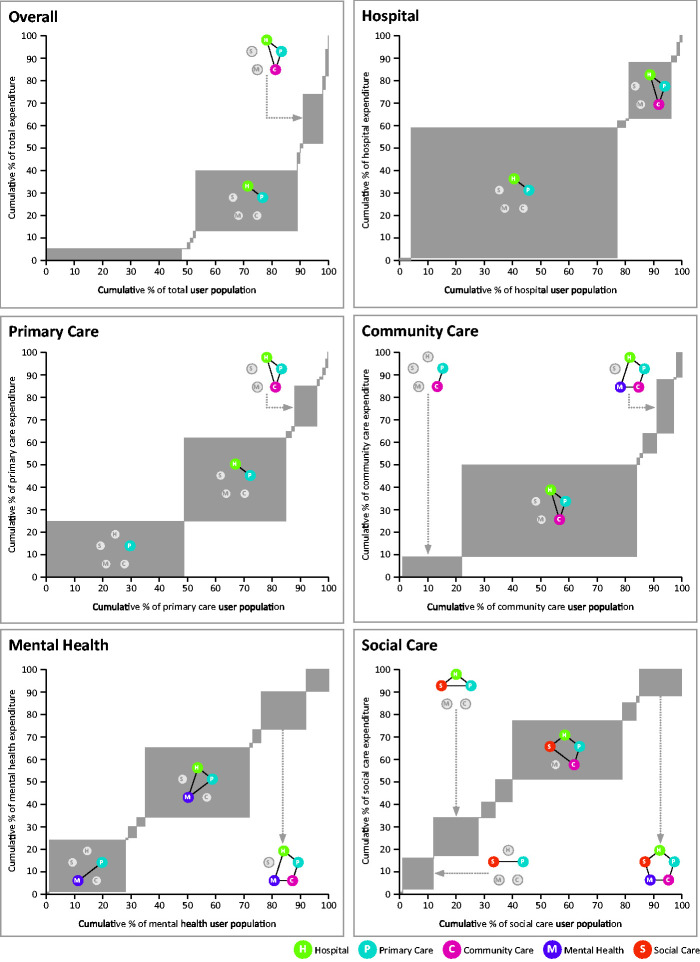
Proportion of total expenditure by proportion of service user population, overall and by setting of care, for each combination of service use.For each graph, the width of the grey box represents the percentage of the population that use that combination of services, and the height of the box shows the percentage of the total expenditure. If a box is wide but not high, it is a high proportion of the population but a small proportion of expenditure (service use). If the box is high but not wide, it is a high proportion of expenditure but a small proportion of the population. Only those combinations with large surface area have been labelled.

Of the 32 possible combinations of service use, the most common combinations as it relates to population volume and proportion of total expenditure were primary-hospital care (27% of total expenditure, 30% of the resident population) and primary-hospital-community care (21% and 6%). This is further illustrated in Figure 1, which shows which service use combinations are most dominant in each setting of care and overall. Eight further service use combinations were identified as being dominant proportions of the expenditure and user volume for individual settings. Primary care was present in all combinations. For mental health, we identified three groups which constituted substantial proportions of overall mental health expenditure but were not dominant when looking at the whole system. For example, the hospital-primary-mental health combination accounted for 37% of the mental health service user population and 31% of total mental health expenditure, but only 1% of the total user population and 5% of the total expenditure.

**Figure 2.**
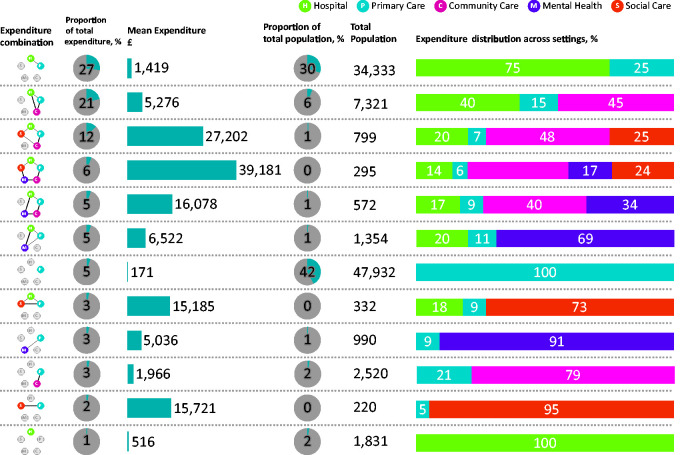
A summary of the 12 expenditure combinations that had more than 150 service users.

**Figure 3.**
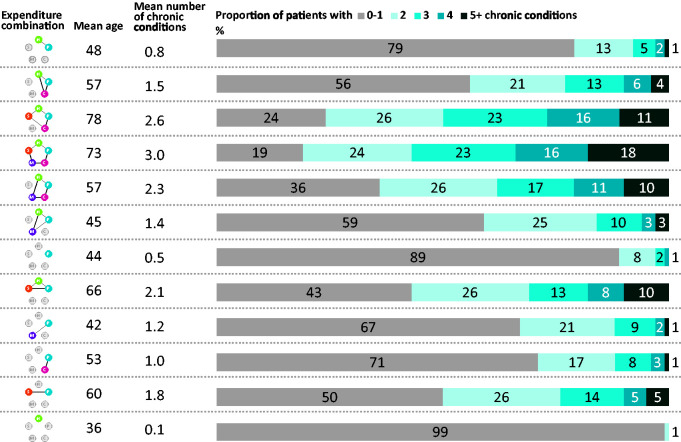
The mean age and number of chronic conditions for the 12 expenditure combinations that had more than 150 service users.

Only 12 of the 32 possible combinations of service use had more than 150 service uses. For each of these 12 combinations, Figure 2 shows the proportion of expenditure, mean expenditure and distribution of expenditure across settings. It also shows the proportion and total number of the population that account for the servicer user combination. Only 0.3% (n = 295) of the population incurred expenditure in all five settings of care (Figure 2, row 4). This population tended to be older, with a mean age of 73, and had higher levels of multi-morbidity with an average of three chronic conditions (Figure 3, row 4). The mean age and the mean number of chronic conditions increased as the number of settings increased. Likewise, mean expenditure increased substantially as the number of settings increased as shown in Figure 2. Community care was a dominant proportion of the mean expenditure for combinations that included 4 or 5 settings of care, accounting for between 39 and 48% of the total mean expenditure. Combinations that included mental health had a lower mean age compared to combinations that did not include mental health. They also had a lower number of chronic conditions. Where there was mental health service use, mean expenditure increased substantially (Figure 2). For example, the hospital-primary care combination incurred a mean cost of £1419, while the expenditure in the hospital-primary care-mental health service combination was, at £6522, almost five times higher. Expenditure in four settings without mental health services was, at £27,202, lower than in five settings with mental health services (£39,181) by a factor of 1.4.

## Discussion

This study demonstrates the potential for large, linked datasets to provide a deeper level of understanding of service use patterns across settings of care in one local authority in London, UK. We found that almost half of total expenditure (47%) in 2016/17 was incurred by community care, social care and mental health services. Further, while mental health service use accounted for just over a tenth of all expenditure in the borough, using mental health services substantially increased mean expenditure per patient. This highlights the need for decision makers and practitioners interested in understanding costs of service use to look beyond primary care and hospital services and set these in the context of wider service use.

Primary care was common to almost all setting combinations. This is consistent with the role of primary care as first contact care, serving, in the UK context, as gatekeeper to other services.^19^ People with multiple conditions attend general practice more than any other NHS service and rely on primary care to coordinate their care.^20^ For primary care, there was a large proportion of activity that was not linked to other settings. It is important to recognise the proportion of primary care patient contacts that do not include liaising with other services when designing integration programmes and engaging the primary care community.

Integration of physical and mental health services is a common concern in most health care systems.^21^ In our study, users of mental health services were, on average, younger and had fewer chronic conditions than users of other services. However, the addition of mental health to service use combinations increased mean expenditure. While age and morbidity levels are associated with higher service use in other settings, we did not find that to be the case for mental health.

Age segmentation and chronic condition counts can enable health systems to identify the small proportion of high users (and by inference those with high needs) who account for a large proportion of total expenditure, but it risks over-prioritising those with existing high needs rather than those with emerging needs. In addition, it does not provide clarity on how specific patient journeys and utilisation patterns can be influenced and altered or which settings of care need to be engaged to implement the changes. Overall, our analysis shows that a large proportion of expenditure was incurred outside of hospital. The use of person-level data allowed us to investigate the proportion of people that use each combination of services and the scale of that service use (level of expenditure).

In England, the potential for integration has been centred on the wide variation in avoidable use of hospital care, and the need to reduce fragmentation and improve experience for people using multiple services by increasing care in the community.^22^ We did not assess the level of integration. And while the highest volume of expenditure was in primary and hospital services, the highest mean expenditure was for a small proportion of service users, those that used all five settings of care.

### Strengths and weaknesses

The main strength of this study was that it included five settings of care and used a large population cohort, which can enable a deeper understanding of patient flows.

There are several limitations. Firstly, data was drawn from a single financial year and longitudinal patterns were not evaluated. Assessing how the service utilisation varies over time provides an interesting avenue for further research. Secondly, by defining multimorbidity as simple count of chronic conditions, our analysis weighted all conditions equally, although the effect of multimorbidity on individuals can vary with combination and severity of conditions. Thirdly, we did not take account of the duration of chronic conditions, which could change the pattern of service use as, for example, the diagnostic pathway and first year of living with a condition can require different service use than subsequent disease management.

While the cohort was large, it was located in one local authority area in east London which is characterised by high levels of deprivation. This may impact generalisability of findings, particularly given known associations between deprivation and increased prevalence of illness and multi-morbidity^23^ and increased service use.^24^

## Conclusion

Using linked electronic health and council data, this study provides insights into service use patterns across settings of care in one metropolitan area in England. These insights will be most useful to practitioners and decision makers seeking to understand differences in use of different types of services and associated costs, which may help inform service planning and strategies to integrate services across the care continuum. Our findings suggest that a whole-system perspective across all settings of care can improve understanding of service user patterns. However, setting-level analysis remains important as there are populations that constitute dominant proportions of the volume and expenditure profile of an individual setting that are not seen at the aggregate whole-system level, in particular mental health users.

## Supplemental Material

sj-pdf-1-hsr-10.1177_1355819620936721 - Supplemental material for Understanding health and care expenditure by setting – who matters to whom? Click here for additional data file. Supplemental material, sj-pdf-1-hsr-10.1177_1355819620936721 for Understanding health and care expenditure by setting – who matters to whom? by Jenny Shand, Stephen Morris and Manuel Gomes: on behalf of the NORTHSTAR-study group in Journal of Health Services Research & Policy
