# Supplementary material for: Understanding health and care expenditure by setting – who matters to whom?
Source: J Health Serv Res Policy. 2020 Jun 30;26(2):77–84. doi: 10.1177/1355819620936721 (PMC8013836; doi:10.1177/1355819620936721)
Supplement: sj-pdf-1-hsr-10.1177_1355819620936721 - Supplemental material for Understanding health and care expenditure by setting – who matters to whom? [file sj-pdf-1-hsr-10.1177_1355819620936721.pdf]

Online Supplement

Overview of the Barking and Dagenham data resource

In 2017, the leaders of Barking and Dagenham Council, North East London NHS Foundation Trust (NELFT) and Barking and Dagenham, Havering and Redbridge Clinical Commissioning Group (BHR CCG), and their Caldicott guardians (a senior person within each organisation who is responsible for protecting the confidentiality of people's health and care information and making sure it is used properly), signed data sharing agreements to create a linked dataset for the population of Barking and Dagenham (B&D) between 1st April 2011 and 31st March 2017.

The dataset contains socio-demographic characteristics, health variables, household variables and data on health and social care service utilisation. It is hosted in the Barking and Dagenham, Havering and Redbridge NHS Accredited Data Safe Haven and contains routinely collected, retrospective, pseudonymised data. It was created for research purposes with ongoing governance and oversight provided by the Barking and Dagenham, Havering and Redbridge Information Governance Steering Committee. The dataset is not currently publicly available, but there is agreement to update the dataset on an annual basis and make it available to the wider research community in autumn 2020. The figure below provides an overview of the dataflows.

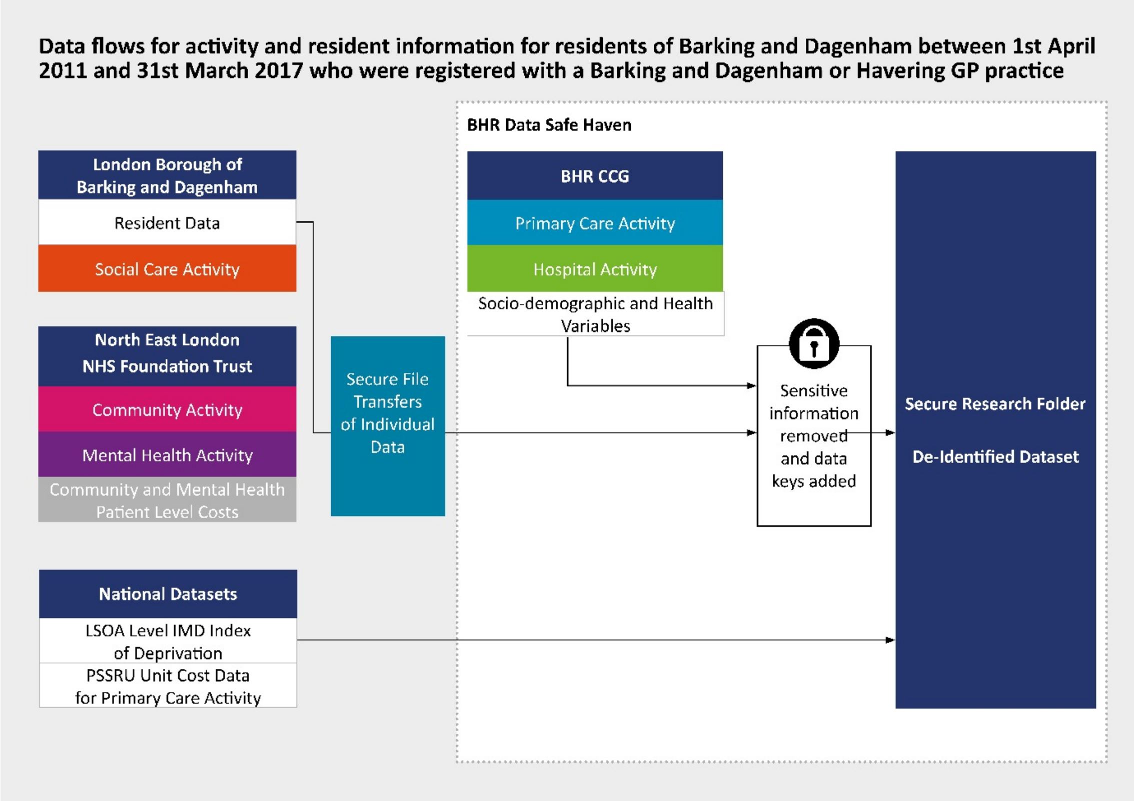

The dataset contains the following variables:

Variable	Description	Values	Source
**Socio-demographic characteristics**			
Age	Age group of the individual	19-49, 50-64, 65-74,	Primary care records

Variable	Description	Values	Source
		75-84, 85+	
Gender	Whether the individual is Male or Female	Male, Female	Primary care records
Ethnic Group	The ethnic group of the individual, applying the ONS ethnicity groupings to the ethnicity documented in the primary care record	White, Black or Black British, Mixed, Other, Asian or Asian British, Unknown	Primary care records
Carer	Whether the individual is a carer, has a carer or both	None, Has a carer, Is a carer, Has and is a carer	Primary care records
**Health variables**			
BMI Category	The BMI category of the individual, assigned using the BMI value recorded in the primary care record	Underweight, Healthy, Overweight, Obese, Morbidly obese	Primary care records
Smoking category	The smoking status of the individual, assigned using the smoking status recorded in the primary care record	Non-Smoker, Ex-Smoker, Smoker, Unknown	Primary care records
Count of Long Term Conditions	The count of how many of the 16 conditions listed below the individual has been diagnosed with	0-1, 2, 3, 4, 5+	Primary care records
Conditions	Whether or not the individual has been diagnosed with each of 16 conditions: Atrial Fibrillation, Asthma, Cancer, CHD, COPD, Dementia, Depression, Diabetes, Epilepsy, Heart Failure, Hypertension, Hypothyroidism, Learning Difficulty, Mental Health, Palliative Care, Stroke	1 if they have been diagnosed with the condition, 0 if not	Primary care records
**Household variables**			
Benefits	If the individual is in receipt of housing benefit, council tax benefit or both, the additional benefits they are also in receipt of	None, Employment Support Allowance (ESA), Pension, Income Support, Job Seekers Allowance, Standard	B&D council housing department
Tenure	The legal status under which people have the right to occupy their accommodation, provided from council records	Owner occupied, private rented, social housing, unknown	B&D council housing department
Occupancy	The number of people living in the household, provided by council records	1, 2 to 4, 5 to 7, 8 to 10, 11+	B&D council housing department
B&D IMD quintile	The quintile in which the overall score from the 2015 index of Multiple Deprivation (IMD) should be assigned to, rebased to Barking and Dagenham quintiles, from Office of National Statistics and applied to the Lower Super Output Area of the address of the individual	1 (least deprived), 2, 3, 4, 5 (most deprived)	National dataset
**Outcome measures: Service Utilisation**			

Variable	Description	Values	Source
Total expenditure	The sum of individual level expenditure across hospital, primary care, community health, mental health and social care	Numeric value	Calculated in the dataset
Hospital expenditure	The sum of individual level expenditure for emergency department, elective and non-elective inpatient activity and outpatient activity	Numeric value	Hospital Episode Statistics (HES)
Primary care expenditure	The sum of individual level expenditure for GP contacts, non-GP contacts and prescriptions	Numeric value	Primary care records
Community Health expenditure	The sum of individual level expenditure for community service activity	Numeric value	North East London NHS Foundation Trust, the local community provider
Mental Health expenditure	The sum of individual level expenditure for inpatient and outpatient mental health activity	Numeric value	North East London NHS Foundation Trust, the local community provider
Social Care expenditure	The sum of individual level expenditure for social care activity	Numeric value	B&D council, adult social care department

The analysis for this paper used cost-weighted utilisation by setting and overall. The expenditure was estimated from activity data, with different methods used for the different settings of care. The total expenditure for the financial year was calculated for each individual by aggregating expenditure across five settings of care: hospital, primary care, community, mental health and social care.

Hospital

Activity was collated across four domains of hospital care: Emergency department attendances, Elective inpatient stays (including day cases), Non-elective inpatient stays (including people admitted but staying for less than 24 hours) and Outpatient attendances.

The dataset included the Healthcare Resource Group (HRG) national tariff assigned to each unit of activity, using the HRG grouper and national tariff in place for the 2016/17 financial year(1). The costs used were reflective of the true cost to the commissioner of the activity. The tariff for each **emergency department** attendance varied depending on the type of emergency department (consultant-led emergency departments; consultant-led mono-specialty services; other types of minor injury departments; and NHS walk-in centres), whether the patient was admitted or not, and whether they arrived at the emergency department by ambulance. These adjustments were made by the HRG grouper and the national tariff for individual level activity reflected these adjustments. The national tariff for **elective inpatient** stays varies according to the specialty of the department, the type of admission, diagnostic tests and procedures performed during the inpatient stay and the overall length of stay for the patient. **Non-elective** cases are assigned different tariffs for short stays (less than two days) and long stays (two days or longer). **Outpatient attendance** tariffs alter depending on the specialty of the department and agreed thresholds of new to follow up ratios. There was activity that had no cost attached to it, for example outpatient visits that exceeded the tariff trim point. This activity was therefore not reflected in the total hospital expenditure

Primary care

Our activity data from primary care records provided the monthly count of attendances with a General Practitioner (GP), with a non-GP, and the number of prescriptions for each individual. Unit costs from the 2016/17 Unit Cost Health and Social Care were used for GP attendances(2). The report calculates costs for GP services by comparing salary, overheads, and other costs for the practice to perform the activities taking into account how long each activity takes. We used the 2016/17 unit cost of £38 per visit, which includes direct staff costs, qualification and training, for a surgery consultation with a GP. This assumes attendances are on average 9.22 minutes in duration. The activity data provided a count of the number of attendances an individual had per month with a "Non-GP". Non-GP attendances may be with a practice nurse, pharmacist or health care assistant. Unit costs from the 2016/17 Unit Cost Health and Social Care for GP practice nurses were used, which was £42 per hour with qualifications. It was assumed that a nurse would see four people per hour, providing a unit cost of £10.50 per visit, and as with the GP attendances no distinction was made between face to face and telephone consultations. The local prescription data provided total prescription costs per practice and total number of prescriptions per practice for 2016/2017. This allowed for a unit cost per prescription to be calculated for each practice and applied to calculate prescription costs for each patient within that practice.

Community services

Activity data was extracted from the RiO system of the local community provider, NELFT. This provided information on the referral source and primary reason for referral, the cost centre the activity was assigned to (to facilitate matching of activity to unit costs), the type of consultation (new or follow up), location of the care contact (e.g. clinical setting or individuals home), whether it was in person or a telephone consultation. Each of these impacted the unit cost of the activity.

Data from the patient level information and costing system from NELFT was used to assign unit costs for each component of activity. This was calculated by the trust by distributing the budget within each department across the activity of the department. Unit level costs were then provided, by department for each type of activity (new or follow up, in person or by phone, location of care contact).

Mental health

As with community services, activity data was extracted from the RiO system of the local community provider, NELFT. This provided information on the referral source and primary reason for referral, the cost centre the activity was assigned to (to facilitate matching of activity to unit costs to the appropriate department), the type of consultation (new or follow up), location of the care contact (e.g. clinical setting or individuals home), whether it was in person or a telephone consultation. Each of these impacted the unit cost of the activity. Data from the patient level information and costing system from NELFT was used to assign unit costs for each component of activity.

Social care

Local authority social care costs were obtained from council data which lists the billed cost for each care package per week for each care recipient. This provided the granularity on in year changes to packages and the resultant change in package costs. Weekly packages included crisis intervention, home care, supported living placements, residential and nursing home placements. Data on self-

funded social care was not available, neither was equipment, transport and home adaptation costs as these are held in different council departments.

References

1. Monitor. 2016/17 National Tariff Payment System. NHS England; 2016.
2. Curtis LA, Burns A. Unit Costs of Health and Social Care 2017. 2017.

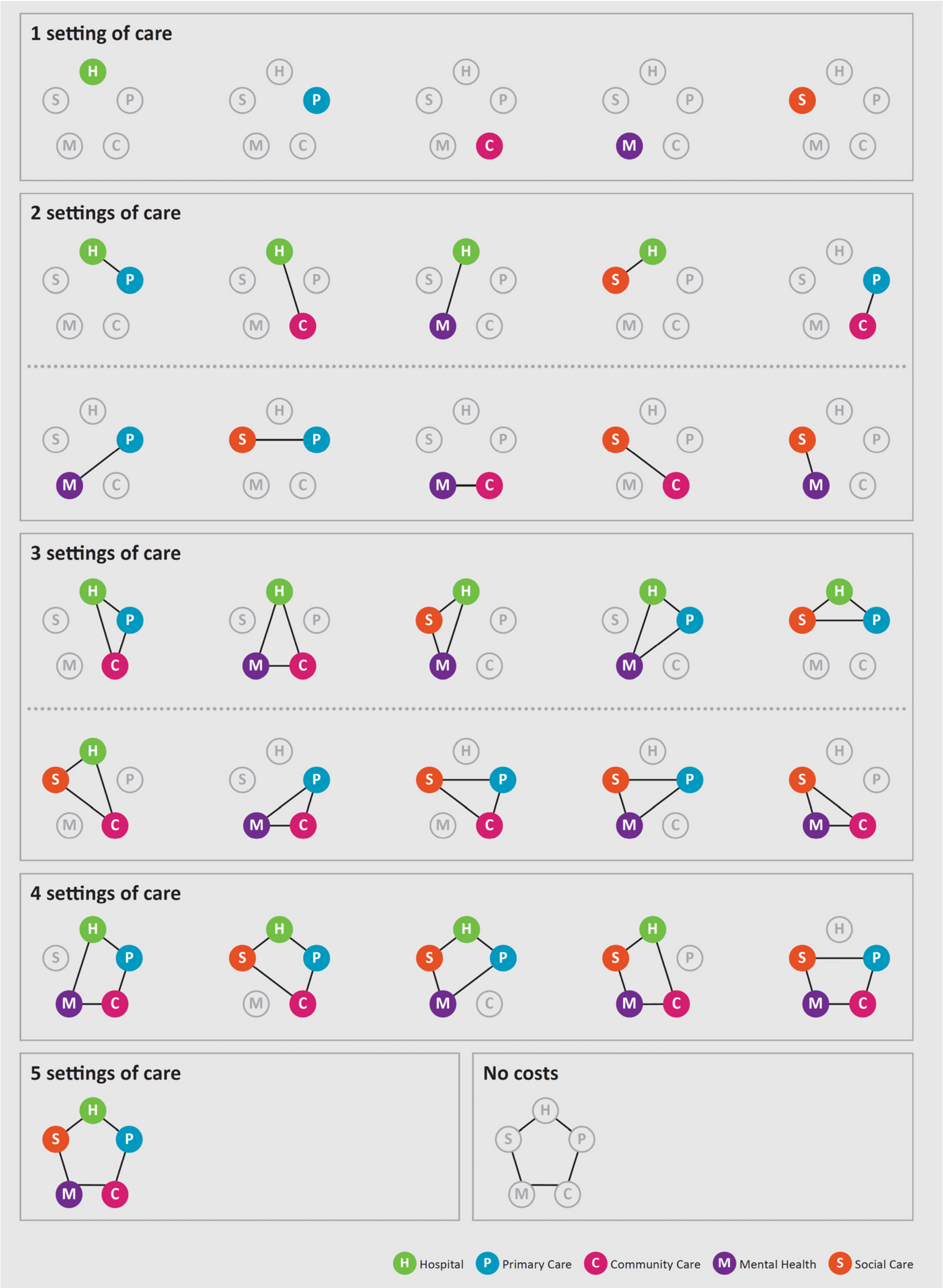

Figure A.1 Combinations of service use (expenditure) across five settings of care
